# Psychiatric Comorbidity: Occurrence and Treatment

**Published:** 1994

**Authors:** Norman S. Miller

**Affiliations:** Norman S. Miller, M.D., is an associate professor at the departments of psychiatry and neurology, University of Illinois, Chicago, Illinois

## Abstract

Alcoholics with psychiatric disorders often fail to receive adequate treatment. Integrated treatment programs show promise for helping these patients.

Comorbidity is the occurrence of two or more illnesses in the same person. The term “psychiatric comorbidity,” as used in this article, refers to the occurrence in the same person of at least one mental disorder and one addictive disorder. For example, a person suffering from schizophrenia may be addicted to alcohol and perhaps to other drugs as well.^1^

Psychiatric comorbidity presents problems for clinicians and patients alike. Comorbid disorders are poorly understood and frequently misdiagnosed, and their treatment is problematic. Alcoholics with psychiatric disorders often fall through the cracks in the health care system, failing to receive treatment for one or the other of their disorders (Minkoff 1989; Ries 1993). Nevertheless, prospects for effective treatment are improving with the increasing integration of psychiatric and addiction treatment perspectives. This article explores some problems in recognizing comorbidity and evaluates current treatment models.

## Evaluating Psychiatric Comorbidity

Epidemiologic studies attempting to measure prevalence rates for psychiatric comorbidity have produced widely varying results, as discussed below. To evaluate and interpret these results, the following factors must be considered: population selection, the perspective of the researcher or clinician, definitions and diagnostic criteria, and followup.

### Population Selection

Study subjects may be drawn from the general population or from patients in treatment (Lyons and McGovern 1989; Pepper et al. 1981; Miller and Fine 1993). Higher rates of comorbidity generally are found in patient populations than among the general population. This probably is because persons with multiple disorders are among the most likely to seek treatment (Pepper et al. 1981). Estimates of the type and prevalence of comorbidity in a patient population may reflect the primary type of treatment those patients are receiving. For example, a general psychiatric hospital would contain more chronically mentally ill (CMI) patients (e.g., schizophrenics and manic-depressives) than would be found ordinarily at an alcoholism treatment center. Moreover, prevalence rates for comorbidity (i.e., chronic mental illness together with alcohol and other drug [AOD] disorders) are higher for inpatient than for outpatient treatment settings and greater for public (i.e., community) than for private settings.

### Perspective of the Researcher or Clinician

The clinician’s or researcher’s perspective is a key factor in assessing prevalence rates. Psychiatrists tend to emphasize psychiatric explanations for alcoholism, whereas alcoholism treatment professionals emphasize alcoholism as an independent disorder (Schuckit 1985). These biases affect the way populations are selected and defined and how disorders are diagnosed.

### Definitions and Diagnostic Criteria

Overestimates of psychiatric comorbidity may result from failure to distinguish between symptoms and disorders. For example, alcoholics undergoing prolonged periods of alternating intoxication and withdrawal frequently manifest symptoms such as hallucinations, paranoid delusions, and thought disturbances. These symptoms occur in disorders such as schizophrenia and mania but also can be induced by alcohol consumption in the absence of any psychiatric disorder. Similarly, alcohol consumption can produce symptoms of anxiety and depression in patients who do not have independent anxiety or depressive disorders. Thus, the frequency of psychiatric symptoms in alcoholics is much higher than the occurrence of true psychiatric disorders.

Confusion of symptoms and disorders can be avoided by the careful use of appropriate diagnostic criteria. The *Diagnostic and Statistical Manual of Mental Disorders, Fourth Edition* (DSM–IV) is a standard guide to defining and diagnosing mental and addictive disorders. In many cases, the DSM–IV provides exclusionary criteria to help distinguish between AOD-induced symptoms and independent disorders. For example, the criteria for diagnosing a major depressive episode stipulate that the “symptoms are not due to the direct physiological effects of a substance (e.g., a drug of abuse, a medication)...” (American Psychiatric Association 1994, p. 327). Currently, the exclusionary criteria tend to be applied more stringently in addiction treatment settings than in psychiatric settings (Miller and Fine 1993).

### Followup

Perhaps the only way ultimately to establish the prevalence of true psychiatric comorbidity in a population is to observe the subjects longitudinally (Miller et al. 1991; Schuckit 1985). Such studies consistently have found that alcohol-induced symptoms of depression and anxiety disappear within several weeks after cessation of drinking without specific treatment (Miller et al. 1991; Schuckit 1985). Psychiatric symptoms that persist may represent independent disorders and, hence, true comorbidity.

## Estimates of Comorbidity

An important source of comorbidity data is the Epidemiologic Catchment Area (ECA) program of the National Institute of Mental Health. The ECA surveyed more than 20,000 persons across the United States to obtain data on the lifetime occurrence of psychiatric disorders (Regier et al. 1990). This study reported lifetime prevalence rates of 19.8 percent for any addictive disorder among persons with a mental disorder, 55 percent for any mental disorder among persons with alcohol disorders, and 64.4 percent for any mental disorder among persons with other drug disorders (Regier et al. 1990).

Conclusions about psychiatric comorbidity based on these data are problematic, in part because the data were obtained retrospectively through interviews with no followup. Moreover, the study defined alcohol-use disorders to include the isolated occurrence of sufficient symptoms, often years apart, to meet the overall criteria for the disorder (Helzer and Pryzbeck 1988).

Various studies have reported high comorbidity rates among psychiatric patients. Specifically, AOD disorders were found among 30 percent of those psychiatric patients appearing to have depressive disorders, 50 percent of those appearing to have manic-depressive disorders, 50 percent of those with schizophrenic disorders, 80 percent of those with antisocial personality disorder, 30 percent of those appearing to have anxiety disorders, and 25 percent of those appearing to have phobic disorders (Brady et al. 1991; Regier et al. 1990; Drake and Wallach 1989; Pepper et al. 1981).

In contrast to the above, prevalence rates for psychiatric disorders among patients under treatment for AOD disorders are considerably lower: 5.0 percent for depressive disorders, 0.8 percent for manic-depressive disorder, 1.1 percent for schizophrenic disorders, 2.0 percent for antisocial personality disorder, 3.0 percent for anxiety disorders, and 6.0 percent for phobic disorders (Helzer and Pryzbeck 1988). These rates generally are similar to those found in an ECA-based survey of the general population in three cities (Myers et al. 1984).

Despite the conflicting assumptions employed in these studies, it can be concluded that substantial psychiatric comorbidity exists, especially for schizophrenia, mania, and antisocial personality disorder (Schuckit 1985; Drake and Wallach 1989; Lehman et al. 1989). Based on ECA data, alcoholics are 21.0 times more likely also to have antisocial personality disorder compared with nonalcoholics. In addition, alcoholics are 6.2 times as likely to have mania and 4.0 times as likely to have schizophrenia, but they are only 1.7 times as likely to have a major depressive disorder and essentially no more likely to have an independent anxiety disorder (Helzer and Pryzbeck 1988).

## Current Approaches to Treating Comorbidity

Three approaches have been developed for treating patients with comorbid AOD and psychiatric disorders. These may be described as the serial, parallel, and integrated treatment models.^2^

### Serial Treatment Model

The traditional approach to comorbidity treatment is the serial model, in which AOD and psychiatric disorders are treated consecutively. Patients who are actively psychiatrically ill are excluded from addiction treatment units. Therefore, in the serial model, a patient is first stabilized at an inpatient or outpatient psychiatric unit, often with the use of medications. Subsequent addiction treatment is conducted by different staff in different locations. The staffs treating each type of disorder do not communicate or cooperate in the patient’s treatment and are often mutually antagonistic in their approach. For example, psychiatric staff often provide explanations for the patient’s alcoholism that contradict those provided by the addiction counselors (Ries 1993).

Serial treatment relies on referrals between the psychiatric and addiction treatment systems. Such referrals, however, have not been common. In particular, CMI patients rarely are transferred to addiction settings, because they often do not blend in well with the more mentally and emotionally stable AOD patients (Minkoff 1989; Ries 1993).

The serial model is not so much a treatment concept as a default situation. Thus, the failure to transfer CMI patients to addiction settings may be seen not as a disadvantage of the serial model per se but as a failure of the health care system to employ any model at all.

### Parallel Treatment Model

In the parallel model, psychiatric and addiction treatment are provided concurrently but still by separate staff in different settings. The parallel model works best when the different settings are located in the same hospital or clinic, maximizing cooperation between disparate staff. When this is not possible, the patient must commute between treatment centers and be treated by staff that are “foreign” to one another. This experience can be difficult on vulnerable patients, such as the CMI, who find such change to be stressful. The dropout rates from addiction or psychiatric treatment can be high in the parallel model (Minkoff 1989; Ries 1993).

### Integrated Treatment Model

The integrated model combines methods and skills derived from both psychiatric and addictive treatment practices (Minkoff 1989). Because each staff member is trained in the treatment approaches of both fields, the diagnosis and treatment for both psychiatric and addictive disorders often can be carried out simultaneously, and conflicts between the two approaches are minimized (Minkoff 1989).

A major limitation of the integrated model is the tendency to undertreat addictive disorders and overtreat psychiatric disorders in patients who have sought treatment for the psychiatric consequences of their addiction (Minkoff 1989; Ries 1993).

The integrated model is suitable for truly comorbid patients, such as the AOD-addicted CMI. Patients who require relatively intensive psychiatric care, such as those who are schizophrenic, manic, or who have severe personality disorders, can better utilize the less intensive addiction program typical of the integrated model.

Few studies have assessed the outcome of comorbidity treatment. Hoffman and colleagues (1993) studied an integrated program treating multiple-addicted patients, most of whom had long histories of psychiatric symptoms as well.^3^ Treatment was described as a modified therapeutic community, in which patients discussed one another’s behavior patterns openly in a group setting. This confrontational approach was found useful for some patients who tended to deny their maladaptive behaviors. However, many patients, particularly the CMI, were unable to tolerate the intense interpersonal interactions generated by such a system.

Therefore, a second track was developed for the CMI patients, consisting of a smaller, more relaxed group that emphasized practical addiction education and the learning of social skills (Hoffman et al. 1993). Both CMI and non-CMI patients were hospitalized in the same inpatient ward. Treatment outcome was evaluated 3 months after discharge on the basis of five variables: abstinence; employment; compliance with prescribed medications; regular attendance at treatment appointments; and the occurrence of major untoward events, such as divorce or arrest.

No differences in treatment outcome were found between the CMI and non-CMI groups. Abstinence was approximately 70 percent for both groups and would have been higher, except that some patients who had lapsed from abstinence for as little as 1 day—or one drink—were classified as nonabstinent. These results suggest that integrated treatment is feasible for groups of patients on the same ward who have multiple addictive and psychiatric disorders and a wide range of functional impairment.

Drake and colleagues (1993) assessed 4-year outcomes for 18 schizophrenic alcoholics treated in an innovative outpatient program. The program used an integrated approach with drug addiction treatment specifically tailored for people with severe chronic mental disorders. Following discharge, each patient received intensive continuing care, including weekly visits with a case manager, antipsychotic medications, and housing and other social supports. At 4 years after discharge, more than one-half (61.1 percent) of the subjects had achieved stable remission from alcoholism. The mean duration of remission was 26.5 months.

## Summary

Estimates of comorbidity vary widely, depending in large part on the clinician’s or researcher’s perspective and the setting in which treatment occurs. These same factors often determine how a patient is diagnosed and what type of treatment is provided. Alcoholics with psychiatric disorders who are caught between conflicting treatment approaches sometimes fail to receive any treatment. Nevertheless, there is an increasing tendency toward integrating addictive and psychiatric treatment. Preliminary studies of two such integrated programs demonstrated success rates comparable with those for alcoholism treatment in general, thereby providing hope that co-morbid psychiatric disorders need not prevent effective addiction treatment.

## Footnotes

1 The term “alcohol addiction” is equivalent to “alcoholism,” which varies according to the reference cited. Also, see glossary for definitions of psychiatric illnesses mentioned in this article.

2 The specific use of medications in treating comorbidity is discussed in the article by Anton, pp. 265–271.

3 Diagnoses were not finalized until the end of the first month of hospitalization to distinguish between AOD-induced symptoms and underlying mental disorders.

## Glossary

Antisocial personality disorder (ASPD): A pervasive pattern of disregard for, and violation of, the rights of others. The disorder is characterized in part by risk-taking, criminality, and pathological lying.
Anxiety disorders: A group of disorders characterized by unrealistic fear, panic, or avoidance behavior. These disorders include (among others) panic attacks, phobias, obsessive-compulsive disorder, and generalized anxiety disorder.
Depressive disorders: A group of disorders including various forms of depression and manic-depression.
Generalized anxiety disorder: A disorder characterized by at least 6 months of persistent and excessive anxiety and worry not resulting from exposure to a drug or medication.
Major depressive episode: A period of at least 2 weeks during which there is either depressed mood or the loss of interest or pleasure in nearly all activities.
Mania: A period of abnormally and persistently elevated, expansive, or irritable mood.
Manic-depressive: Characterized by alternating manic and depressive episodes.
Mood disorders: Depressive and manic disorders.
Panic disorder: A disorder characterized by sudden, unexpected, and persistent episodes of intense fear, accompanied by a sense of imminent danger and an urge to escape.
Phobia: A marked and persistent fear of specific objects or situations.
Posttraumatic stress disorder (PTSD): A disorder often brought on by major emotional trauma such as sexual or physical assault. It is characterized by the reexperiencing of the traumatic event (in dreams, illusions, or hallucinations) and by avoidance of stimuli associated with the trauma.
Schizophrenia: A chronic disorder characterized by delusions, hallucinations, and disorganized thought and behavior.
